# Remarks on Vesico-Vaginal Fistule

**Published:** 1856-11

**Authors:** N. Bozeman

**Affiliations:** Montgomery, Alabama


*Additional Remarks on Vesico-Vaginal Fistule and the comparative value of the Clamp and Button Satures.* By N. BOZEMAN, M. D., of Montgomery, Alabama.

IN some editorial remarks which recently appeared in the American Medical Gazette, and the Nashville Medical and Surgical Journal, a statement which I made in an article on Vesico-Vaginal Fistule, published in the May number of the Review in regard to the success of the clamp suture in the treatment of this affection, is not only called in question, but absolutely asserted to be unfounded. This statement was, in effect, that, to the best of my knowledge, the clamp suture of Dr. Sims has failed in as many trials as it has succeeded, or, in other words, that in a given number of operations—not cases—the clamp suture fails in one half. The effect of a positive contradiction from two such sources, although not substantiated by any reference to statistics or facts, being to place me in an unpleasant attitude before the profession, I am compelled in justice to myself to explain the grounds upon which my opinion was based.

I have no means of knowing any thing about Dr. Sims' practice since he left this city, nor do I wish to disparage it in the least. I can only speak of what I saw and knew of his success previous to his removal to New York. For three or four years I assisted him in many of his operations, and witnessed their results. There are also several other physicians in this community who saw even more of his operations than I did; and not one, I venture to say, will deny that I did, in the statement referred to, more than full justice to Dr. Sims.

No one, I presume, who reads my paper in this journal with ordinary care, could fail to discover that my object in bringing the button suture before the profession, was simply to present a comparison of its results with those of other methods. The statement as to the clamp suture was made in a general way. I referred to its employment by no one in particular excepting myself. I need scarcely say, this was done from personal friendship to Dr. Sims. I was sufficiently acquainted with the results of his practice, to know that they would not compare favorably with what I had obtained by the button suture, and I could then have produced the same facts in support of this assertion that I am now forced to make public. It appears that the statement referred to, is considered as applying to Dr. Sims alone. As it has been placed upon that footing, I am perfectly willing that it should stand so. I shall be abundantly able to prove, if it becomes necessary, that *not one half* of his individual operations before his removal to New York, were successful.

It will suffice for my present purpose to allude to those cases in which I applied the button suture with entire success, after Dr. Sims' suture had failed in his own hands. The report of two of these, (cases I. and II,) is appended to my former article. The former, I mentioned, had been operated upon *once*, and the latter *several times* according to the method of Dr. Sims. Case II., was under his treatment for more than three years, and I am satisfied he performed upon it not less than ten or a dozen operations, several of which I assisted in myself. Upon another case, (Mrs. H., of Auburn, Ala.,) whom I have just cured, he operated twice.

My own experience with the clamp suture amounts to eight operations, six of which were complete failures. Two of these operations were performed upon a case in which Dr. Sims had met with no less than five or six failures. The case was discharged uncured. There are other cases within my knowledge, upon some of which Dr. Sims performed so many operations that the patients themselves have no recollection of the number. Such is my knowledge of the results of the clamp suture, such the foundation upon which I rested the opinion that the number of its successes did not exceed the number of its failure.

As additional support to the advantage claimed for the button suture, I may here state that since the preparation of my paper referred to, I have performed nine other operations, making in all, sixteen. Of this number, only one partial failure has occurred. This was my twelfth operation, and resulted solely from carelessness on my part, in not giving the button a proper shape. Several of these cases were very interesting. In one, the fistulous opening was about an inch in length, and complicated with a rent in the anterior lip of the cervix uteri. Both fistule and rent were closed at one sitting. This is the second case, with this complication, which has come under my observation ; the other I operated upon last year, and may be found reported in the Southern Med. and Surg. Journal for Aug. 1855. So far as my knowledge extends, this is the first case of the kind which has been recorded. Dr. Sims informed me that he had performed the same operation shortly after my report appeared. In another one of these cases, the fistulous opening was nearly an inch and a half in its transverse diameter, and the anterior lip of the cervix formed its posterior border.

The most remarkable case, however, was one in which nearly the whole of the septum had sloughed out. The opening was somewhat of a quadrangular shape, and so large that the superior fundus of the blader protruded through and appeared at the vulva in the form of a large fleshy tumor. It measured transversely an inch and three quarters, and longitudinally about an inch and a half. The anterior lip of the cervix uteri formed, as in the preceding case, the posterior border of the chasm. Both ureters were exposed to view and seemed to open on the vaginal side of the septum, which appearance resulted from an eversion of the edges of the fistule. The procedure adopted here was to pare thoroughly the edges, and then slit both ureters on the vesical side of the septum to the extent of a quarter of an inch. The object of this was to throw the entrance of the urine into the bladder away from the approximated edges of the fistule. This having been done, the cervix uteri was next brought down and secured to the anterior border of the opening which was immovable. The button used in this instance was semicircular in shape and nearly two inches in length. The sutures, eight in number, formed parts of the radii of a circle whose centre was about half an inch above the os uteri. In no case could the result have been more satisfactory, and a most interesting circumstance was that the cervix and part of the body of the uterus were made to close the fistulous opening.

The stiches in both of these cases were taken in the anterior lip of the cervix, just as though it had been part of the vesico-vaginal septum. I am not aware that an operation similar to these has ever been performed by any one else in this country—at least I have never seen one reported. As to applying the clamp suture in either one of these cases, it would have been utterly impossible with any reasonable hope of success.—*Louisville Review.*

